# Brain network topology early after stroke relates to recovery

**DOI:** 10.1093/braincomms/fcac049

**Published:** 2022-02-22

**Authors:** Paul R. Nemati, Winifried Backhaus, Jan Feldheim, Marlene Bönstrup, Bastian Cheng, Götz Thomalla, Christian Gerloff, Robert Schulz

**Affiliations:** Department of Neurology, University Medical Center Hamburg-Eppendorf, 20246 Hamburg, Germany; Department of Neurology, University Medical Center Hamburg-Eppendorf, 20246 Hamburg, Germany; Department of Neurology, University Medical Center Hamburg-Eppendorf, 20246 Hamburg, Germany; Department of Neurology, University Medical Center Hamburg-Eppendorf, 20246 Hamburg, Germany; Department of Neurology, University Medical Center, 04103 Leipzig, Germany; Department of Neurology, University Medical Center Hamburg-Eppendorf, 20246 Hamburg, Germany; Department of Neurology, University Medical Center Hamburg-Eppendorf, 20246 Hamburg, Germany; Department of Neurology, University Medical Center Hamburg-Eppendorf, 20246 Hamburg, Germany; Department of Neurology, University Medical Center Hamburg-Eppendorf, 20246 Hamburg, Germany

**Keywords:** severe impairment, diffusion MRI, structural connectome, graph theory, motor recovery

## Abstract

Analyses of alterations of brain networks have gained an increasing interest in stroke rehabilitation research. Compared with functional networks derived from resting-state analyses, there is limited knowledge of how structural network topology might undergo changes after stroke and, more importantly, if structural network information obtained early after stroke could enhance recovery models to infer later outcomes. The present work re-analysed cross-sectional structural imaging data, obtained within the first 2 weeks, of 45 acute stroke patients (22 females, 24 right-sided strokes, age 68 ± 13 years). Whole-brain tractography was performed to reconstruct structural connectomes and graph-theoretical analyses were employed to quantify global network organization with a focus on parameters of network integration and modular processing. Graph measures were compared between stroke patients and 34 healthy controls (15 females, aged 69 ± 10 years) and they were integrated with four clinical scores of the late subacute stage, covering neurological symptom burden (National Institutes of Health Stroke Scale), global disability (modified Rankin Scale), activity-related disability (Barthel Index) and motor functions (Upper-Extremity Score of the Fugl-Meyer Assessment). The analyses were employed across the complete cohort and, based on clustering analysis, separately within subgroups stratified in mild to moderate (*n* = 21) and severe (*n* = 24) initial deficits. The main findings were (i) a significant reduction of network’s global efficiency, specifically in patients with severe deficits compared with controls (*P* = 0.010) and (ii) a significant negative correlation of network efficiency with the extent of persistent functional deficits at follow-up after 3–6 months (*P* ≤ 0.032). Specifically, regression models revealed that this measure was capable to increase the explained variance in future deficits by 18% for the modified Rankin Scale, up to 24% for National Institutes of Health Stroke Scale, and 16% for Barthel Index when compared with models including the initial deficits and the lesion volume. Patients with mild to moderate deficits did not exhibit a similar impact of network efficiency on outcome inference. Clustering coefficient and modularity, measures of segregation and modular processing, did not exhibit comparable structure–outcome relationships, neither in severely nor in mildly affected patients. This study provides empirical evidence that structural network efficiency as a graph-theoretical marker of large-scale network topology, quantified early after stroke, relates to recovery. Notably, this contribution was only evident in severely but not mildly affected stroke patients. This suggests that the initial clinical deficit might shape the dependency of recovery on global network topology after stroke.

## Teaser

Nemati *et al*. report that global efficiency of structural brain networks, quantified directly after severe stroke, positively relates to recovery. Compared with models which include initial deficit and lesion volume, the consideration of network properties led to an increase in explained variance of up to 24%.

## Graphical Abstract

**Figure:**
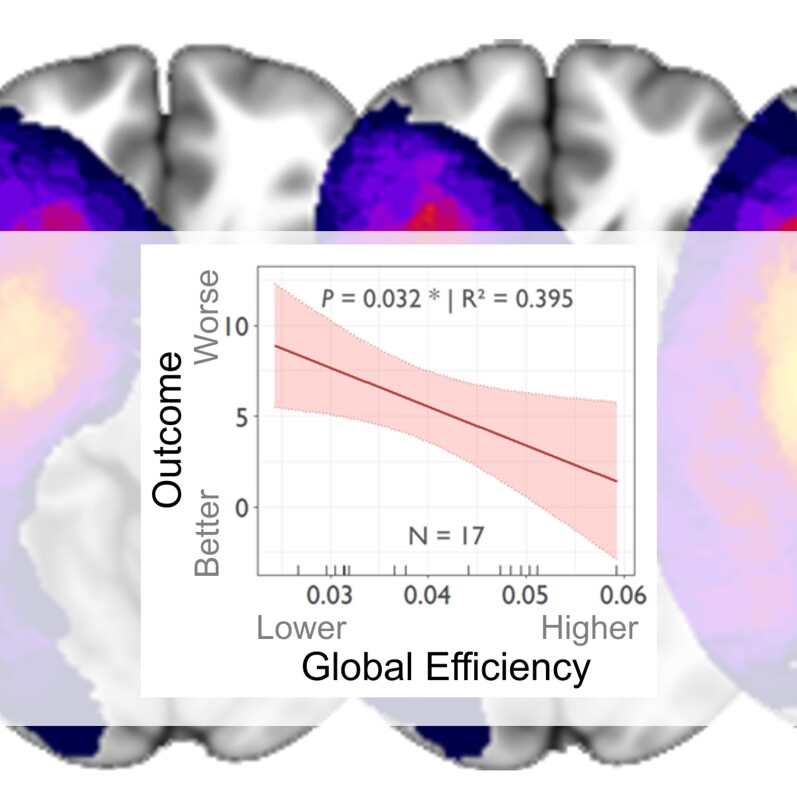

## Introduction

Over the past decade, analyses of complex brain networks have gained an increasing interest in imaging-based stroke and neurorehabilitation research.^1–4^ For instance, in motor stroke research, previous structural investigations have primarily focused mostly on selected brain regions such as the primary motor cortex, important white matter pathways such as the corticospinal tract and, even more recently, on selected long-range corticocortical motor connections that connect frontal and parietal key areas of the human motor network.^5^

There is growing evidence that stroke-related deficits are unlikely to result only from focal brain injury, but that inter-subject variability in persistent deficits and recovery processes are likely to be consequences of direct and indirect effects of lesions onto functioning of distributed brain networks including multiple regions and interconnecting pathways as well.^2,6^ Fundamental concepts of brain network topology^7,8^ emphasize the importance of functional or structural *integration* across and *segregation* between brain areas with aspects of *modular processing*. In the healthy brain, these properties are well-tuned to ensure proper information processing within local modules and to optimize communications across spatially segregated subnetworks via large-scale interactions. Frameworks for network analyses, such as graph theory, have been developed and widely employed to capture network topology in health and disease.^9–14^

Various electrophysiological and imaging studies have explored the interrelationship between alterations of network topology and deficits after stroke.^4,15–21^ Despite the methodological discrepancy between structural and functional connectivity analyses in brain networks, such studies have convergingly evidenced that acute stroke lesions lead to reduced graph measures of network *integration*, such as global efficiency (GE). In contrast, network *segregation* and parameters of *modular processing* are reported to increase after stroke [e.g. measured by clustering coefficient (CC) and network modularity (MOD), respectively]. Resting-state functional MRI has revealed that the gradual re-instatement of normal MOD associates with the functional improvement of language, spatial memory and attention deficits over time.^19^ Insights into structural brain networks topology are remarkably limited.^17,22^ One recent study has demonstrated that, like functional networks over time, also structural brain networks became less integrated and more segregated with decreasing GE and increasing MOD. Herein, greater change in topology was associated with larger residual symptom burden and greater motor impairment over 1 year of follow-up.^17^

Despite these valuable insights into the interrelationship between stroke-related alterations of whole-brain networks and clinical phenotypes, there is only very limited data supporting the view that the consideration of such network data, obtained already early after stroke, might help to enhance predictive models of the subsequent outcomes. In one study, structural topology in the acute stage did not improve correlative models to infer future deficits in 30 stroke patients after 1, 3 or 12 months.^17^ As one limitation, patients included were relatively well recovered in the acute stage with a median National Institutes of Health Stroke Scale (NIHSS) score of only 3.^17^ One resting-state functional MRI study found that network efficiency, obtained acutely after stroke, was positively related to the amount of motor recovery within 3 months. However, limitations of this study included the small sample size (12 patients) and the inclusion of patients with ischaemic infarctions and intracranial haemorrhages.^23^ More recently, other resting-state studies have reported that lower lesion load to strategic network areas was related to improved recovery of overall symptom burden^24^ or of cognitive deficits after stroke.^25^ Also these cohorts consisted of patients with rather mild to moderate symptom load with averaged NIHSS scores between 2^25^ and 5.^24^ Other observational resting-state studies have not investigated the additive value of their network configuration data in outcome inference analyses in detail.^19,20^ Finally, one important limitation of such functional network analyses is, at least in part, its dependency on arousal states^26^ or psychoactive medications,^27,28^ which are often altered or used in acute or early subacute stroke patients. Hence, structural network data might exhibit their particular potential in the clinical setting as imaging biomarkers unbiased by patient alertness or acute stroke medication.

In summary, there is still limited data, which suggest that information regarding individual network topology after stroke might help to infer recovery. The evidence that, e.g. structural network information obtained early after stroke by means of individual whole-brain tractography can actually enhance models to infer later outcome is scarce. Moreover, previous studies have mostly included patients with rather mild deficits. The impact of an altered network topology for outcome inference in severely impaired patients remains largely unknown. The detection of a categorically differential importance of early network characteristics for outcome models might have important consequences for future study designs, including clinically relevant prediction models in larger cohorts. The aim of the present study was to investigate whether graph-theoretical markers of large-scale network topology early after first-ever ischaemic stroke might enhance correlative models to infer subsequent persistent deficits in the late subacute stage of recovery. Specifically, we sought to address this research question in a large cohort of stroke patients with variable deficits and subgroups with mild and severe initial deficits.

## Materials and methods

### Participants and clinical assessment

The present study is based on patient data from previously published cohorts of acute stroke patients of two independent studies. Cohort 1 (C_1_) comprised a total of 61 acute ischaemic stroke patients admitted to the University Medical Center Hamburg-Eppendorf which were recruited from June 2012 to September 2017 in the framework of the Collaborative Research Centre 936. This cohort has already been introduced in detail by our previous reports.^17,29^ In Cohort 2 (C_2_), 30 initially more severely impaired acute stroke patients, admitted to the same medical centre from October 2017 to February 2020, were included. Detailed inclusion and exclusion criteria are given in our recent report on parietofrontal functional connectivity.^30^ In brief, inclusion criteria for both studies were: first-ever unilateral ischaemic stroke, upper extremity motor deficit involving hand function, no history of previous neurological or psychiatric illness, age ≥18 years. In both cohorts, acute stroke patients underwent T_1_-, T_2_- and diffusion-weighted MRI in the first days after the event as time point T_1_ (C_1_: Days 3–5, C_2_: Days 3–14). The follow-up time point T_2_ was defined in the late subacute stage of recovery^31^ after 3 months, or, as in seven cases of cohort C_2_ in which clinical data for this time point was not available, after 6 months.^30^ Across both cohorts, clinical assessments included scores of neurological symptom burden (NIHSS, range 0–42), global disability (modified Rankin Scale, mRS, range 0–6), activity-related disability [Barthel Index (BI), range 0–100] and motor functions (Upper-Extremity Score of the Fugl-Meyer Assessment, UEFM, range 0–66). Data of healthy participants, similar in age and gender, were also re-analysed from both cohorts. All participants provided informed written consent themselves or via a legal guardian, following the ethical Declaration of Helsinki. The original studies were approved by the local ethics committees. For this secondary exploratory analysis, available imaging and clinical data of both cohorts were included according to the following inclusion criteria: supratentorial stroke lesion, availability of MRI data obtained at T_1_ and at least one clinical assessment measured at T_2_.

### Imaging acquisition

For both datasets, brain imaging was performed with a 3 T Skyra MRI scanner (Siemens, Erlangen, Germany). A 32-channel head coil was used to obtain high-resolution T_1_- and T_2_-weighted and diffusion-weighted images (DWIs). For the T_1_-weighted sequence, a three-dimensional magnetization-prepared rapid gradient echo sequence was used. Parameters were: repetition time (TR) = 2500 ms, echo time (TE) = 2.12 ms, flip angle 9°, 256 coronal slices with a voxel size of 0.8 mm × 0.8 mm × 0.9 mm, field of view (FOV) = 240 mm. T_2_-weighted images were acquired by using a fluid attenuated inversion recovery sequence with the following parameters: TR = 9000 ms, TE = 86 ms, TI = 2500 ms, flip angle 150°, 43 transversal slices with a voxel size of 0.7 mm × 0.7 mm × 3.0 mm, FOV = 230 mm. T_2_-weighted images were acquired in order to help delineating stroke lesions. For DWI, an echo planar imaging sequence was used and the whole brain was covered with gradients (*b* = 1500 s/mm^2^) applied along 64 non-collinear directions with the following parameters: TR = 10 000 ms, TE = 82 ms, flip angle 90°, 75 axial slices with a voxel size of 2 mm × 2 mm × 2 mm, FOV = 256 mm. One *b*_0_ image was also collected.

### Image processing and analysis

Individual stroke lesions were delineated using a semi-automatic algorithm in ITK-SNAP version 3.8.0^32^ based on visual inspection of the T_1_-weighted and the DWIs. T_2_-weighted images were also inspected to support the identification of lesioned areas. Pre-processing and reconstruction of raw MRI data were performed with q-space imaging QSIprep, version 0.9.0.^33^ This pipeline performs standardized and reproducible pre-processing of diffusion MRI. First, brain extraction (ANTs’ *antsBrainExtraction*) and tissue segmentation (FSL’s *fast*) of the T_1_-weighted images were performed. Then, DWIs were pre-processed by de-noising the DWI volumes, performing bias field correction and creating a brain mask from the *b*_0_ image. Subsequent steps included corrections of head-motion, eddy currents (FSL’s *eddy*) and susceptibility distortions (FSL’s *topup*). Finally, the *b*_0_ image is co-registered rigidly to the T_1_-weighted image (ANTs’ *antsRegistration*). The pre-processing outputs are the starting point for reconstruction of white matter fibre tracts by using a preconfigured reconstruction workflow offered by QSIprep (*mrtrix_singleshell_ss3t*). In brief, this workflow first creates a template consisting of five tissues (white matter, cortical grey matter, subcortical grey matter, pathological tissue and cerebrospinal fluid) based on the segmented T_1_-weighted image. Importantly, lesion segmentations were not included in this workflow, but used to determine lesion volumes (LVs). This template serves as the prerequisite for identifying anatomical constraints to improve biological plausibility of the streamlines that are created by tractography, e.g. by terminating streamlines before entering cerebrospinal fluid or initiating streamline creation at the boundary between white and grey matter. This leads to an anatomically constrained tractography.^34^ The workflow uses a constrained spherical-deconvolution (CSD) algorithm, a version of a multi-shell multi-tissue CSD algorithm^35^ adjusted for single-shell DWI data for estimation of fibre orientation distribution in the white matter, which is subsequently used for tractography of 10 million streamlines. After that, the streamline weights of the resulting tractogram, which are determined by the count of connecting streamlines, are calculated by applying the spherical deconvolution informed filtering of tractograms 2 method to enhance biological plausibility of tractograms.^36^ To construct structural networks, those modified weights were further included in a structural connectivity matrix derived from the cortical parcellation of the T_1_-weighted image according to the automated anatomical labelling (AAL) atlas.^37^ This atlas segments the brain into 41 cortical, 4 subcortical and 13 cerebellar regions per hemisphere resulting in a total of 116 regions. Hence, the structural connectivity matrix used in this study had the dimension of 116 × 116. Structural connectomes remained un-thresholded, in line with recent suggestions.^38^

### Graph-theoretical network analysis

The Brain Connectivity Toolbox for MATLAB^14^ (brain-connectivity-toolbox.net) was used to compute different graph-theoretical measures of network topology based on un-thresholded, weighted, undirected and normalized (range of connection weights 0–1 according to minimum and maximum) connectivity matrices with self-connections set to zero. Based on clear *a priori* hypotheses derived from our previous study,^17^ we primarily focused on GE and MOD, measures of *network integration* and *modular processing*, respectively. GE is a measure of network integration and is defined as the inverse of the average shortest path length across all nodes of the network^39^ with lower shortest path lengths corresponding to higher global efficiencies of the network and thus more efficient information transfer between distributed brain regions. In contrast, MOD describes the prevalence of distinct modules with a maximally possible number of within-module links and a minimally possible number of between-module links within the network.^40^ MOD values were further processed by computing the means across all 116 nodes of the AAL atlas. In order to provide a comprehensive picture of network topology after stroke, secondary measures included characteristic path length (CPL) and also CC, a surrogate of *network segregation*. Moreover, as un-thresholded dense weighted undirected structural networks were constructed, also network density (*D*) was included to provide information regarding *network sparsity*. All graph-theoretical measures did not undergo further modifications before incorporation into the regression models.

### Statistical analysis

Statistical analyses were carried out in R version 4.0.3 (r-project.org). To discriminate the patient cohort into two subgroups of patients with mild to moderate and severe initial impairment, a *k*-means cluster analysis based on NIHSS and UEFM scores at T_1_ was computed (R’s *k-means*). The rational for this approach was to guide the group discrimination by direct measures of the neurological state after stroke which are robust to potential confounders such as dehydration or infections which may critically impact early disability scales. Between-group comparisons (complete stroke cohort, STROKE; mild to moderate impairment, MILD; severe impairment, SEVR; healthy controls, HCs) of network measures were performed by estimating linear models with the respective measure as the dependent variable (DV), GROUP as the factor of interest and AGE as a nuisance variable. LV and age were also compared between groups by additional models, group-wise with GROUP as the factor of interest. To examine potential associations of early structural network measures with LV in stroke patients, linear models were fit to STROKE, SEVR and MILD with the structural network measures serving as the DV. Log-transformed LV and AGE were treated as covariates.

To describe functional improvement over time from T_1_ to T_2_, linear mixed-effects models with repeated measures (R’s *lmer*) were fit with the four respective outcome scores as the DV, TIME (T_1_, T_2_) as factor of interest and participant as random effect.

To investigate the associations between early network topology at T_1_ with clinical scores at T_2_, we first employed linear models (R’s *lm*) in the complete cohort of stroke patients with NIHSS, mRS, BI and UEFM at T_2_ as DVs in separate models. Network topology measures obtained at T_1_ were tested as the predictors of interest. To address the subgroup-specific importance (MILD/SEVR) of network measures, the interaction term with GROUP was included in extended models. Baseline models also included the specific clinical score at T_1_ and LV (log_10_-transformed) to adjust the target effects. As AGE was not correlated with the initial deficit in STROKE, this factor was not included in the baseline outcome regression models to prevent model inflation, in line with our previous report.^17^ Given significant interactions, subgroup-specific models were fit for MILD and SEVR for the different network and outcome measures. To improve data distribution, NIHSS, BI and UEFM values at T_1_ were transformed by taking the square root in STROKE, but not in SEVR and MILD. Final models were simplified based on the Akaike Information Criterion using R’s *step* function. Initial clinical assessments at T_1_ were always kept in the models. To account for the variability in the precise time point of clinical evaluation (3 or 6 months) used for the follow-up T_2_, SEVR models were tested for the significance of this factor. Model results are given including numbers of patients in the model, estimated coefficients of *z*-standardized predictors with their *P*-values and multiple *R*^2^ of the final model. Statistical significance was assumed at *P* < 0.05. *P*-values of *post hoc* group comparisons between SEVR, MILD and HC were corrected for multiple testing using Tukey tests, otherwise *P*-values are presented uncorrected for multiple comparisons.

### Data availability

Clinical data, imaging data and code used for the analyses are available upon reasonable request from the corresponding author.

## Results

### Demographic and clinical data

The combination of cohorts C_1_ and C_2_ resulted in an initial cohort of 91 stroke patients. Supplementary Fig. 1 gives a flow diagram of data evaluation and analysis. The final analysis contained data of 45 acute stroke patients (22 females, 24 right-sided strokes, age 68.4 ± 12.6 years, mean ± SD). Clustering analysis revealed two sufficiently distinctive subgroups (between sum of squares/total sum of squares ratio = 0.764): one group of 24 severely affected patients (SEVR, 13 females, 14 right-sided strokes, aged 71.2 ± 11.9 years, median NIHSS = 11, median UEFM = 5) and one group of 21 rather mildly to moderately affected patients (MILD, 9 females, 10 right-sided strokes, aged 65.2 ± 12.9 years, median NIHSS = 3, median UEFM = 57). A visualization of the distribution of the NIHSS and UEFM scores used for group allocation is given in Supplementary Fig. 2.

Tables 1 and 2 give the clinical characteristics of SEVR and MILD. Moreover, for HC, data from 34 healthy participants from both initial studies (19 from C_1_, 15 from C_2_, aged 69.3 ± 9.9 years, 15 females) were also analysed. Age did not differ significantly between the groups, neither between STROKE and HC (*P* = 0.730), nor between SEVR, MILD and HC (all *P* ≥ 0.184, Table 3).

**Table 1:** Patient characteristics of SEVR at T_1_ and functional outcome at T_2_

ID	Study cohort	Age	Sex	Lesioned hemisphere/dominance	Thrombolysis/thrombectomy (TICI)	LV (ml)	NIHSS		mRS		BI		UEFM	
							T_1_	T_2_	T_1_	T_2_	T_1_	T_2_	T_1_	T_2_
1	C_1_	43	Male	Right/n	Yes/yes (3)	79.8	13	3	4	2	15	100	4	16
2	C_1_	56	Male	Right/n	No/no	2.5	13	5	4	2	40	85	4	7
3	C_1_	69	Female	Right/n	No/no	7.4	—	10	—	5	—	35	—	5
4	C_1_	49	Female	Left/d	Yes/yes	53.8	10	6	5	2	30	90	4	13
5	C_1_	73	Female	Left/d	No/no	5.8	9	3	4	4	35	50	4	13
6	C_1_	77	Female	Right/n	No/no	9.1	8	2	5	4	30	85	4	—
7	C_1_	65	Male	Left/n	No/no	6.6	8	4	4	3	40	100	6	15
8	C_1_	85	Female	Right/n	No/no	16.7	7	4	4	4	35	65	5	40
9	C_1_	47	Male	Left/d	Yes/no	2.6	6	3	4	3	50	95	6	19
10	C_2_	78	Male	Left/d	No/no	58.1	17	—	5	5*^†^	10	15*^†^	3	—
11	C_2_	83	Female	Left/d	No/no	101.4	20	—	5	6	5	—	6	—
12	C_2_	76	Male	Right/n	Yes/yes (3)	101.0	11	—	5	3^†^	10	—	4	—
13	C_2_	63	Male	Left/d	No/no	55.8	13	1	4	1	40	100	5	36
14	C_2_	77	Female	Right/n	Yes/no	286.7	11	10*	4	4*	20	45*	4	4*
15	C_2_	71	Female	Right/n	Yes/yes (2A)	38.4	9	—	5	3*	10	70*	4	47*
16	C_2_	80	Female	Left/d	No/no	20.5	11	15*	5	4*	0	30*	—	4*
17	C_2_	67	Female	Right/n	Yes/no	7.4	11	7*	4	3*	30	70	6	5*
18	C_2_	80	Male	Right/n	Yes/yes (0)	108.4	16	—	5	6^†^	10	—	6	—
19	C_2_	79	Female	Right/n	Yes/yes (2B)	120.4	8	2*	5	3*	10	85*	15	51*
20	C_2_	85	Female	Right/n	No/yes (2B)	33.5	15	14	5	5	0	0	2	4
21	C_2_	78	Male	Right/n	Yes/yes (3)	178.1	17	3	5	4	10	65	5	15
22	C_2_	76	Male	Right/n	Yes/yes (3)	91.8	15	13	5	4	5	20	6	4
23	C_2_	78	Female	Left/d	No/no	33.6	10	3	4	3	35	80	8	31
24	C_2_	74	Male	Left/d	Yes/yes (2B)	303.3	24	—	5	5*	5	5	5	4*
Mean (SD)		71.2 (11.9)	11 males	14 right/9 days	11 TL/10 TT	71.8 (82.9)	12.3 (4.4)	6.0 (4.5)	4.6 (0.5)	3.7 (1.3)	20.7 (15.3)	61.4 (32.8)	5.3 (2.5)	17.5 (15.7)
Median		76	—	—	—	46.1	11	4	5	4	15	70	5	13

Baseline characteristics and outcome measures are given individually for patients and group-averaged for patients and controls. Assessments took place in the acute stage (T_1_) 3–14 days after the event and in the late subacute stage (T_2_) either 3 or 6 months (depicted by *) after stroke. SD, standard deviation; d, dominant hemisphere; n, non-dominant hemisphere; TICI, thrombolysis in cerebral infarction; TL, thrombolysis; TT, thrombectomy; NIHSS, National Institutes of Health Stroke Scale; mRS, modified Rankin Scale; BI, Barthel Index; UEFM, Upper-Extremity Fugl-Meyer score. For study Cohorts C_1_ and C_2_, see 'Materials and methods’ section. ^†^Follow-up values were taken via phone.

**Table 2:** Patient characteristics of MILD at T_1_ and functional outcome at T_2_

ID	Study cohort	Age	Sex	Lesioned hemisphere/dominance	Thrombolysis/thrombectomy (TICI)	LV (ml)	NIHSS		mRS		BI		UEFM	
							T_1_	T_2_	T_1_	T_2_	T_1_	T_2_	T_1_	T_2_
1	C_1_	62	Male	Left/d	No/no	3.6	3	0	3	1	95	100	37	62
2	C_1_	69	Male	Right/d	Yes/yes (3)	25.1	3	1	4	1	40	100	62	62
3	C_1_	71	Male	Right/n	No/no	0.9	1	0	1	0	100	100	62	65
4	C_1_	65	Male	Left/d	No/no	2.8	3	0	1	1	100	100	65	66
5	C_1_	73	Female	Right/n	Yes/yes (3)	26.8	3	0	4	1	55	100	62	65
6	C_1_	70	Male	Right/n	No/no	5.5	0	0	1	1	100	100	65	66
7	C_1_	81	Female	Right/n	No/no	0.8	1	0	1	0	100	100	65	66
8	C_1_	78	Female	Left/d	No/no	0.8	5	0	1	1	100	100	65	64
9	C_1_	56	Female	Right/n	No/no	1.3	1	1	1	1	95	100	63	65
10	C_1_	49	Male	Left/d	Yes/no	1.8	3	1	2	1	95	100	56	66
11	C_1_	63	Male	Left/d	Yes/no	0.8	3	1	2	1	95	100	42	63
12	C_1_	70	Female	Right/n	Yes/yes	74.4	5	2	4	2	65	95	56	58
13	C_1_	44	Male	Left/n	Yes/yes	85.9	8	4	2	1	85	100	61	66
14	C_1_	78	Female	Left/d	No/no	0.7	0	0	1	1	100	100	66	66
15	C_1_	47	Female	Right/n	Yes/no	7.0	2	0	3	1	90	100	32	66
16	C_1_	54	Male	Left/d	No/no	1.1	5	2	3	1	85	100	41	64
17	C_1_	81	Male	Left/d	No/no	1.7	4	2	4	2	50	85	57	65
18	C_1_	48	Male	Left/d	Yes/yes	24.4	7	1	4	1	25	100	43	65
19	C_1_	87	Female	Left/d	No/no	1.0	1	0	4	1	—	100	32	64
20	C_1_	50	Male	Right/n	Yes/yes	50.1	4	2	4	1	70	100	51	65
21	C_2_	73	Female	Right/n	Yes/yes (2A)	27.6	5	2	4	1	25	80	49	62
Mean (SD)		65.2 (12.9)	12 males	10 right/11 days	10 TL/7 TT	16.4 (25.1)	3.2 (2.2)	0.9 (1.1)	2.6 (1.3)	1.0 (0.4)	78.5 (26.1)	98.1 (5.4)	53.9 (11.6)	64.3 (2.0)
Median		69	—	—	—	2.8	3	1	3	2	92.5	100	57	65

Baseline characteristics and outcome measures are given individually for patients and group-averaged for patients and controls. Assessments took place in the acute stage (T_1_) 3–14 days after stroke and in the late subacute stage (T_2_) 3 months after stroke. SD, standard deviation; d, dominant hemisphere; n, non-dominant hemisphere; TICI, thrombolysis in cerebral infarction; TL, thrombolysis; TT, thrombectomy; NIHSS, National Institutes of Health Stroke Scale; mRS, modified Rankin Scale; BI, Barthel Index; UEFM, Upper-Extremity Fugl-Meyer score. For study Cohorts C_1_ and C_2_, see ‘Materials and methods’ section.

**Table 3:** Group comparison of age, LV and structural network parameters

	STROKE (1)	SEVR (2)	MILD (3)	HC (4)	1–4	2–4	3–4	2–3
	Mean (95% CI)	Mean (95% CI)	Mean (95% CI)	Mean (95% CI)	*P*	*P*	*P*	*P*
*N*	45	24	21	34	—	—	—	—
Age	68.4 (65.0–71.8)	71.2 (66.6–75.8)	65.2 (60.3–70.1)	69.3 (65.4–73.2)	0.725	0.808	0.392	0.184
LV	45.9 (26.5–65.4)	71.8 (45.9–97.7)	16.4 (0–44.1)	—	—	—	—	0.005**
GE	0.0404 (0.0379–0.0429)	0.0393 (0.0358–0.0428)	0.0417(0.0379–0.0454)	0.0461 (0.0431–0.0491)	0.004**	0.010*	0.153	0.637
MOD	3.52 (3.33–3.71)	3.52 (3.25–3.79)	3.52 (3.23–3.81)	3.46(3.24–3.69)	0.710	0.948	0.950	1.000

Estimated means with 95% confidence intervals are given for each group. X–Y indicates the pair of groups for comparison. *P*-values of *post hoc* group comparisons between SEVR, MILD and HC were corrected for multiple testing using Tukey tests. Comparisons of network measures were adjusted for age. LV in ml, MOD, modularity; GE, global efficiency.

* *P* < 0.05.

** *P* < 0.01.

**Figure 1:**
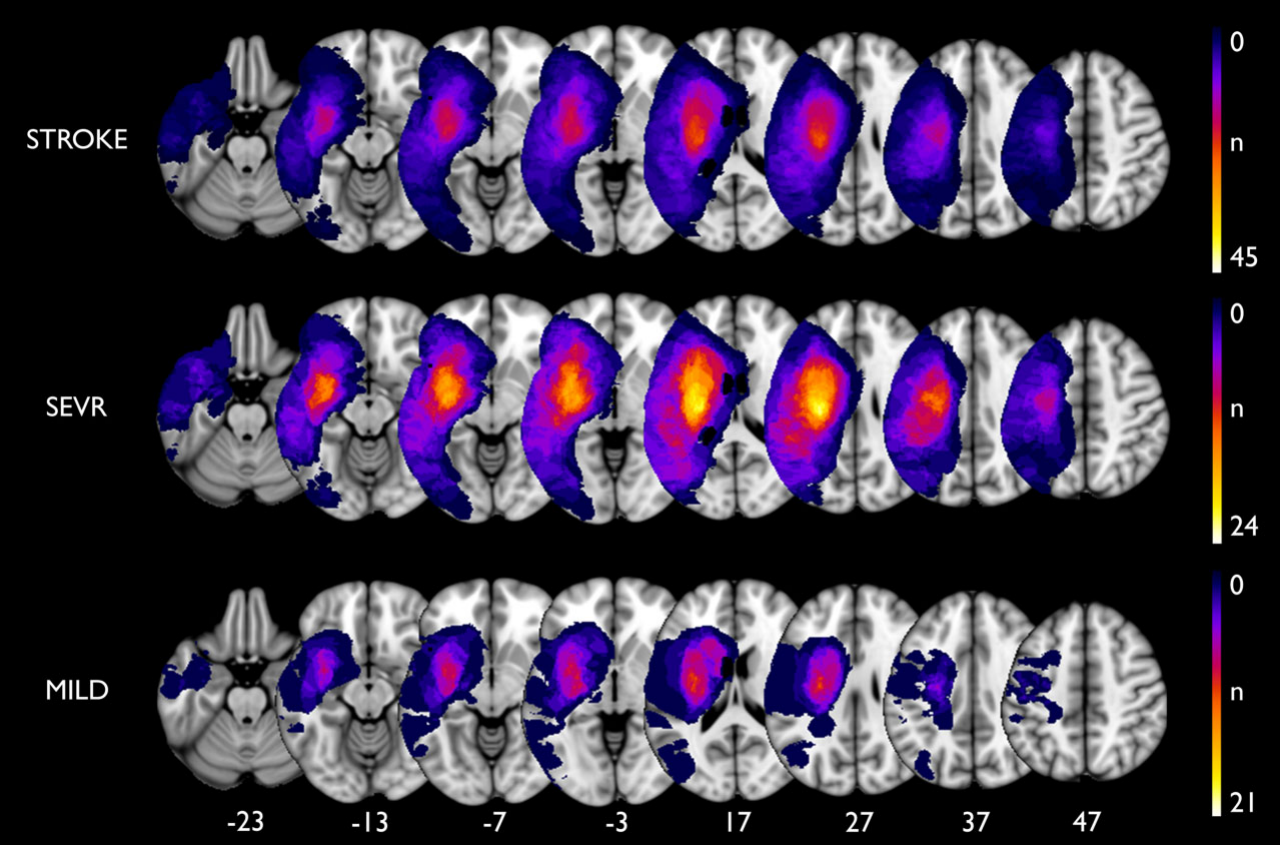
**Lesion topography.** Individual patient lesions are superimposed onto the left hemisphere of the MNI brain (neurological convention). Colour bars indicate the number of patients having their lesions in the respective area. *z*-coordinates of the slices in MNI space are displayed at the bottom.

Fig. 1 depicts the topography of stroke lesions in the STROKE group and both subgroups, respectively. The distribution of LVs across both groups was rather heterogeneous. LVs were larger in SEVR compared with MILD [estimated mean (95% confidence interval), SEVR: 71.8 ml (45.9–97.7), median = 46.1 ml; MILD: 16.4 (0–44.1) ml, median = 2.8 ml; *P* = 0.005, Table 3].

**Figure 2:**
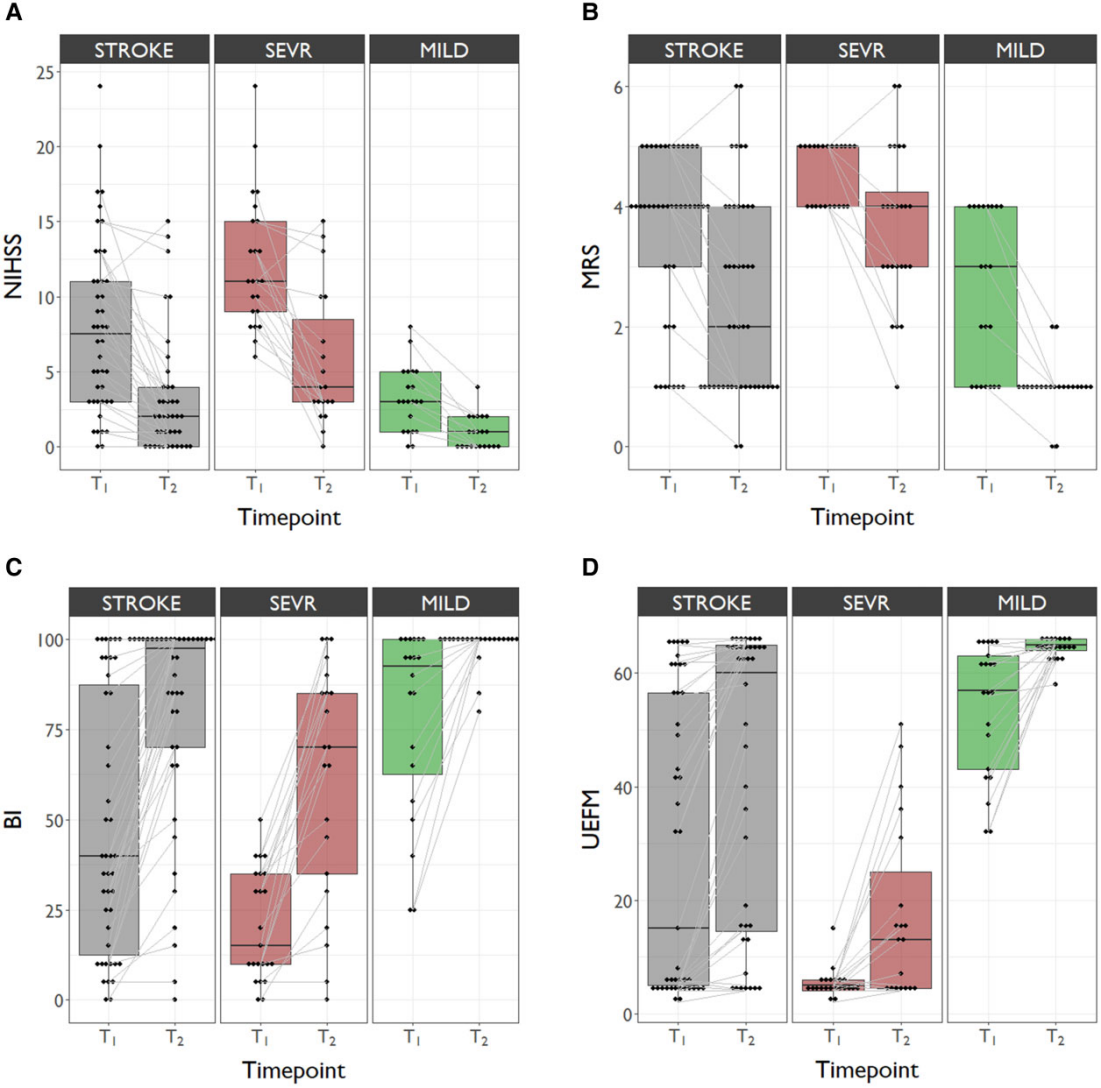
**Functional improvement over time in STROKE, SEVR and MILD.** Boxplots depicting the evolution of NIHSS (**A**), mRS (**B**), BI (**C**) and UEFM (**D**) from T_1_ to T_2_. Linear mixed-effects modelling was used to compare clinical measures within the respective groups. Based on these models, least-squares were subsequently computed with Tukey’s test as the *post hoc* analysis method.

Linear mixed-effects models evidenced significant functional improvement over time from T_1_ to T_2_ regarding all four scores in the complete cohort STROKE (all *P* < 0.001) and also within SEVR (mRS *P* = 0.002, all other *P* < 0.001) and in MILD (NIHSS *P* = 0.015, mRS *P* < 0.001, BI/UEFM *P* = 0.003). Fig. 2 illustrates the evolution of functional scores over time.

### Group comparisons of early structural network topology after stroke

**Figure 3:**
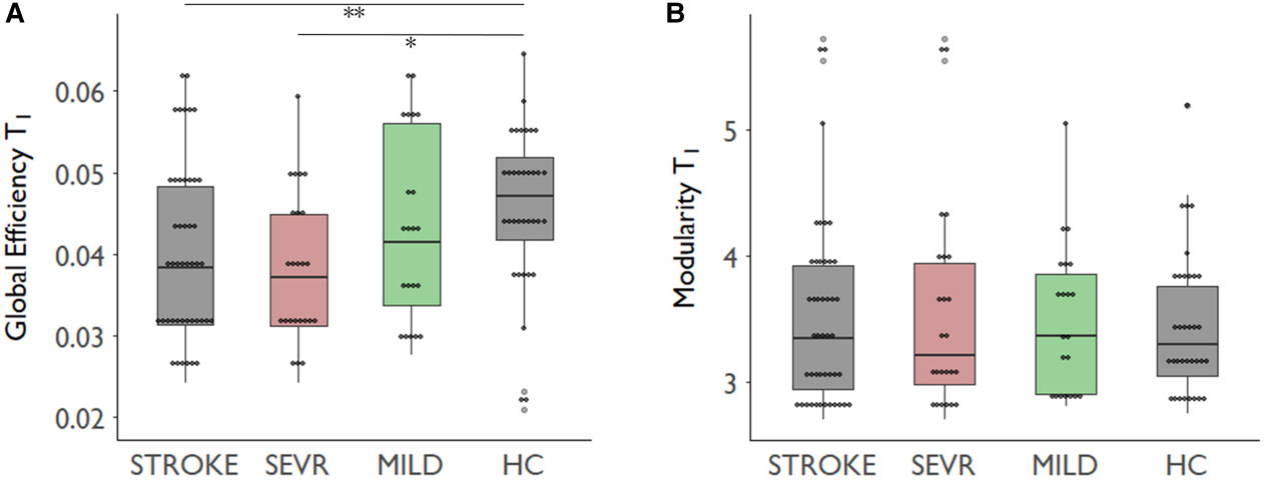
**Structural network topology after stroke.** Boxplots depicting early MOD (**A**) and GE (**B**) for STROKE, SEVR, MILD and HC. Group comparisons were conducted by computing least-squares from linear models including GROUP and AGE with Tukey’s test as the *post hoc* analysis method. *P < 0.05, **P < 0.01.

GE at T_1_ was significantly reduced in STROKE compared with HC (*P* = 0.004, Table 3). Also, the comparison between SEVR, MILD and HC revealed a significant main effect for GROUP (*P* = 0.011), with SEVR showing a significant reduction in GE compared with HC (*P* = 0.010) in *post hoc* testing. In contrast, the numerical reduction of GE in MILD did not reach statistical significance when compared with HC (*P* = 0.153). For MOD, we did not find any significant GROUP effect at T_1_, neither between STROKE and HC (*P* = 0.710) nor between SEVR, MILD and HC (main effect GROUP *P* = 0.933, Table 3). For the latter comparison, *post hoc* pair-wise tests did not yield any group differences for MOD at T_1_ (all *P* ≥ 0.948, Table 3 and Fig. 3). LV did not correlate with MOD and GE, neither in STROKE (all *P* ≥ 0.373) nor in the subgroups SEVR (all *P* ≥ 0.607) or MILD when analysed separately (all *P* ≥ 0.242, Supplementary Fig. 3). Secondary analyses of CC and CPL did not uncover any significant group differences between STROKE, HC, MILD and SEVR. Only for *D*, we found significantly reduced values for STROKE when compared with HC (*P* = 0.004) and for SEVR when compared with HC or MILD (both *P* < 0.001). Data distribution of *D* for the different subgroups is illustrated in Supplementary Fig. 4. In fact, GE and *D* showed a significant positive correlation in all subgroups (*P* < 0.05, not shown), a finding already known from the literature.^41^ Results for CC, CPL and *D* are summarized in Supplementary Table 1.

### Early structural network efficiency and subsequent recovery after stroke

**Figure 4:**
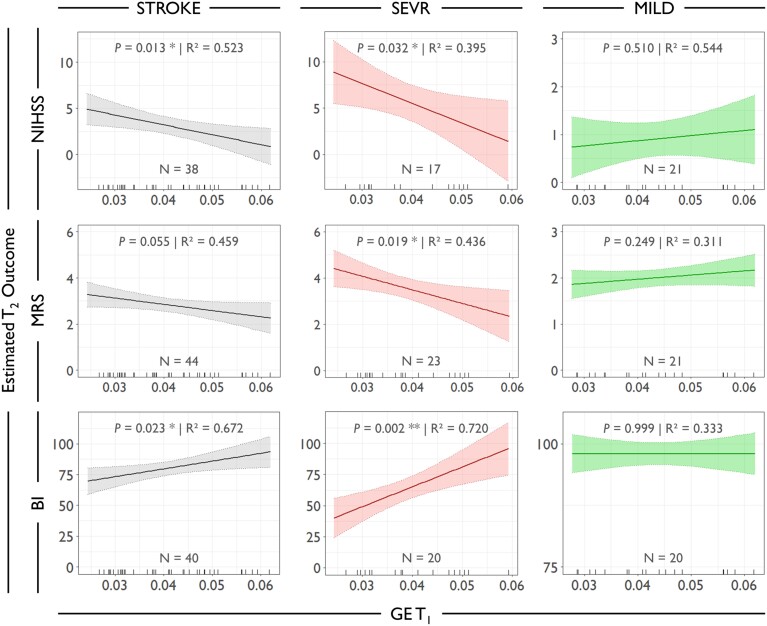
**Early structural network efficiency and subsequent recovery after stroke.** Effect plots showing associations between early GE at T_1_ and the estimated outcome at T_2_ for all stroke patients (STROKE models without GROUP and interaction term). Based on significant GE–GROUP interactions, effect plots are also given separately for SEVR and MILD. *P*-values are given for the predictor of interest GE, multiple *R*^2^ are given for the complete models. *N*, number of patients contributing to the model. *P < 0.05, **P < 0.01.

For the entire cohort of stroke patients (STROKE), regression analyses revealed significant associations between GE at T_1_ and both NIHSS (*n* = 38, *P* = 0.013) and BI (*n* = 40, *P* = 0.023, Fig. 4) at T_2_, after adjustment for the initial functional scores at T_1_. Specifically, higher GE values were associated with lower NIHSS and higher BI values, i.e. lower degrees of impairment. For mRS, we found a similar trend for significance (*n* = 44, *P* = 0.055). For UEFM at the T_2_ follow-up, we did not detect any significant influence of GE. LV did not significantly contribute to these models and was omitted during model simplification.

Next, we explored extended models which included the interaction GE × GROUP to assess any group-specific influence of GE onto functional recovery after stroke. This interaction was significant for NIHSS (*P* = 0.020), mRS (*P* = 0.008) and BI (*P* < 0.001) but not for UEFM. Subsequently, for the former three models, we repeated the regression analyses separately for SEVR and MILD and found that GE exclusively exerted a significant influence on NIHSS (*n* = 17, *P* = 0.032), mRS (*n* = 23, *P* = 0.019) and BI (*n* = 20, *P* = 0.002) in the SEVR but not in the MILD cohort (*n* = 21 for NIHSS and mRS, *n* = 20 for BI, Table 4 and Fig. 4). In the MILD cohort, GE estimates at T_1_ (all *P* ≥ 0.249) did not improve the correlative outcome models, which were dominated by the initial clinical scores. LV did not exert any significant contribution to the final models and was omitted during model simplification. In the MILD cohort, GE was also not correlated with the clinical scores at T_2_ when omitting the initial deficit from the models to enhance residual unexplained variance (all *P* ≥ 0.129, not shown). In other words, the effect in the entire STROKE cohort was driven by the severely affected patients (SEVR). In the SEVR cohort, additionally explained variances of the final models increased by 24.7% for NIHSS, 18.1% for mRS and 19.8% for BI when compared with models, which only included the initial deficit and the LV (not shown). Taking NIHSS at T_1_ into account for mRS and BI models for STROKE and SEVR did not alter the overall findings (Supplementary Table 2). Given significant group differences for *D*, regression models were also estimated for this network parameter. Results were largely in line with the findings for GE and are given in Supplementary Table 3 and Fig. 5. For completeness, modelling results are also given for MOD, CC and CPL which did not show any group differences at T_1_. None of these measures was correlated with clinical scores at T_2_ (Supplementary Tables 4–6).

**Table 4:** Early structural network efficiency and subsequent recovery after stroke

			Model summary			
Outcome	Group	Predictor	Coef.	*P*	*F*	*R*²
NIHSS T_2_	STROKE	GE	−0.61	0.002**	12.47	0.602
		GROUP		0.292		
		GE × GROUP		0.020*		
		NIHSS T_1_	0.44	0.030*		
	SEVR	GE	−0.50	0.032*	4.56	0.395
		NIHSS T_1_	0.38	0.091		
	MILD	GE	0.11	0.510	10.73	0.544
		NIHSS T_1_	0.69	<0.001***		
mRS T_2_	STROKE	GE	−0.53	0.002**	14.29	0.595
		GROUP		0.025*		
		GE × GROUP		0.008**		
		mRS T_1_	0.34	0.027*		
	SEVR	GE	−0.43	0.019*	7.73	0.436
		mRS T_1_	0.46	0.013*		
	MILD	GE	0.23	0.249	4.05	0.311
		mRS T_1_	0.49	0.022*		
BI T_2_	STROKE	GE	0.62	<0.001***	28.36	0.764
		GROUP		0.802		
		GE × GROUP		<0.001***		
		BI T_1_	0.65	<0.001***		
	SEVR	GE	0.48	0.002**	21.80	0.720
		BI T_1_	0.6	<0.001***		
	MILD	GE	<−0.01	0.999	4.24	0.333
		BI T_1_	0.58	0.010**		
UEFM T_2_	STROKE	GE	0.12	0.106	87.62	0.834
		UEFM T_1_	0.89	<0.001***		

Linear models correlating GE at T_1_ with clinical outcome at T_2_. GE × GROUP interactions were evaluated in the whole stroke cohort (STROKE) for NIHSS, mRS and BI. Group-specific models were fit in the case of a significant interaction. For UEFM the non-significant interaction term was omitted from the model (see ‘Results’ section). Model predictors are derived from baseline models and model simplification (see ‘Statistical analysis’ section). Outcome and predictor values were *z*-standardized to enable comparability of coefficients. *R*² given as multiple *R*² of the complete model.

* *P* < 0.05.

** *P* < 0.01.

*** *P* < 0.001.

In order to explore further characteristics of SEVR in the present cohort with respect to the contribution of brain network topology to recovery, we explored NIHSS subitems and tested additional GE models for these subscales (summarized in Supplementary Table 7). SEVR was mainly characterized by deficits in the motor domain, sensory disturbances, dysarthria and extinction/neglect. GE models are summarized in Supplementary Table 8. They indicate that network–outcome relationships for GE might be particularly driven by improvement in the motor domain (NIHSS Items 4–6) and in extinction/neglect (NIHSS Item 11).

Final analyses focused on the relationships between GE, LV and clinical scores at T_1_. Across all patients (STROKE) significant associations were found between LV and NIHSS, mRS and BI, where larger lesions correlated with more impairment, after adjustment for GROUP (all *P* ≤ 0.001). LV × GROUP interactions were not significant. Only UEFM at T_1_ was not related to LV (*P* = 0.973). In contrast to LVs, GE did not correlate with the initial deficits, neither in STROKE (all *P* ≥ 0.376), nor separately in SEVR or MILD as evidenced by non-significant GE × GROUP interactions.

## Discussion

The main findings of the present structural network analyses were (i) a significant reduction in GE, a graph-theoretical measure of *network integration*, early after ischaemic stroke and (ii) a significant negative association of this measure with the extent of persistent functional deficits in the late subacute stage. That is, the higher structural GE was early after stroke (T_1_), the better the clinical outcome on follow-up (T_2_). Importantly, subgroup analyses revealed that this association was driven by patients with more severe initial deficits and not detectable in patients with mild to moderate symptom burden. Measures of *modular processing* and *network segregation* did neither exhibit comparable alterations early after stroke nor did they show network–outcome associations, neither in severely nor in mildly affected patients.

### Early alterations of structural network efficiency after stroke

The present findings extend the results of our previous cohort study, which showed that focal brain lesions lead to increasingly less integrated structural networks over time after stroke.^17^ Our data complement these temporal profiles (i) by showing that the stroke-related decrease in GE is already significant in the early subacute stage and (ii) that this effect is driven by severely affected patients with larger lesions and higher initial symptom load. Under the assumption that lower structural efficiency would reduce the potential to facilitate functional connectivity between brain regions,^42^ this result is well in line with one EEG study on functional networks showing an acute reduction in network integration after stroke.^21^ However, there are also fMRI studies that have not detected any significant changes in network topology acutely after stroke, even in patients with severe deficits.^20^

### Early structural network efficiency relates to recovery after stroke

Previously, it has been evidenced that the amount of increasing dis-integration across networks correlated with the amount of final residual symptom burden. Importantly though, early network topology did not relate to later outcomes which led to the conclusion that *only* the change over time and *not* the absolute values of topology metrics, e.g. obtained in the acute stage, might be informative for structural–clinical relationships.^17^ Although methodologically different to tractography and structural networks, analyses of functional resting-state networks had not corroborated this hypothesis, particularly with respect to the motor domain, one of the most influential factors for sequelae after stroke.^19^ The present study now provides evidence that structural GE, a surrogate of network integration, obtained in the early subacute stage by means of whole-brain tractography, can significantly enhance regression models to explain inter-subject variability in subsequent persistent deficits, operationalized by established scores, such as NIHSS, mRS and BI 3–6 months after stroke. It evidences that already absolute GE, and not *only* its change over time,^17^ might be informative to enhance predictive outcome models. LV as a contributing factor was neither related to GE, nor did it add to infer the functional outcome after stroke, but it was significantly correlated with the initial deficits. GE was not related to the initial deficit. These distinct relations suggest that early GE relates to subsequent recovery processes and is not a surrogate of mere symptom burden after stroke which is further supported by the statistical approach of taking the initial clinical deficit into account. One previous resting-state study found that acute CPL was related to improvement in motor function 3 months after stroke. However, this study did not correct for the initial deficits.^23^ Apart from that study, the importance of early network topology for inference of neurological recovery has only been assessed by few structural^22^ and functional imaging studies.^18,19,24,25^ For instance, the former integrated acute lesion data with normative connectome data to interpolate individual early network disruption and related it to recovery processes. However, individual network topology in older stroke patients might differ significantly from normative topologies, particularly when these data were collected in younger participants and not age-matched controls.^22^

The early decrease in GE and its association with subsequent deficits were only present in patients with more severe deficits. In this subgroup, early GE explained up to 24% in the variability of the clinical outcome when compared with models which included the initial clinical deficit and the LV. In our view, it is important to point out that this finding might have been missed if the entire cohort would have been modelled without assessing the interaction between the initial deficit and the influence of GE on recovery. In that case, one might have come to the erroneous conclusion that GE could explain the outcome in patients with mild to moderate *and* severe deficits. However, as illustrated by the effect plots of Fig. 4, variability in GE in the mildly affected group at T_1_ was largely uncorrelated with the outcome at T_2_. This also held true when omitting the initial deficit from the models in order to increase the variance in the outcome to explain by GE. How can we interpret this difference between patients with severe and mild to moderate initial impairment? Structural imaging had already evidenced that severe strokes with larger LVs lead to more widespread alterations of white matter microstructure of the motor network not only of the ipsilesional but also of the contralesional hemisphere.^43^ Residual motor functions of chronic stroke patients with larger corticospinal tract damage have been reported to additionally depend on the integrity of premotor–motor connections—an association not detected in patients with smaller lesion load.^44^ Functional imaging studies have shown an increasing dependency of residual motor functions on distributed key motor areas of the contralesional hemisphere.^45^ Hence, we propose that the present results might indicate that the extent of the initial impairment can moderate the association between preserved network topology and better outcome after stroke. With regard to future studies, these results might also argue that particularly patients with more severe deficits might be more suitable to study relationships between network topology after stroke and behaviour. Neuroimaging studies are clearly more feasible in stroke patients with less severe deficits. However, this selection bias might hide some relevant mechanisms, as the present data suggest.

### Limitations

There are a number of important limitations worth noting: first, the present analysis is based on the combination of independent samples of two different studies. Models were statistically corrected for this factor; significant contributions were not detected. The same holds true for the combined time point of the outcome measure at T_2_, either after 3 or 6 months.^30^ The additional consideration of this factor in the models did not alter the findings. Second, group allocation was conducted based on a clustering analysis based on NIHSS and UEFM scores. Hence, this allocation should be considered arbitrary; it is specific to the present cohorts and might be different in independent samples. Also, group composition might change when considering alternative approaches of patient stratification, such as the PREP algorithms which combine clinical scores and measures of the structural and functional integrity of the corticospinal tract.^46,47^ Third, the reduced variance in the outcome variables in the mildly impaired patients is an inherent statistical limitation. Most patients in this group showed a favourable recovery until T_2_. However, this limitation is relativized by the finding in MILD that for NIHSS, mRS and BI, the respective score at T_1_ was needed to explain the T_2_-value. As an additional *post hoc* analysis, we adapted the initial models for these three outcome variables and included the interaction GE × Outcome-T_1_ (continuous) instead of GE × GROUP. In other words, we omitted the potentially arbitrary group allocation for each patient in each outcome model. For all GE models, the interactions remained significant (NIHSS *P* = 0.03, mRS *P* = 0.01, BI *P* < 0.001), indicating that the influence of higher GE at T_1_ on better recovery at T_2_ increases with higher initial deficit. Fourth, particularly in SEVR, there were still a lot of missing data at T_2_. Thus final models were fit on reduced sample sizes. Fifth, we found significant associations between GE and NIHSS, mRS and BI, measures of disability and symptom burden, but not UEFM, a measure of motor functions. This might indicate an outcome-specific importance of network topology after stroke. In fact, imaging studies have repeatedly shown that, at least in part, results might be specific to certain outcome measures and not easily generalized across others.^17,30,48^ As an alternative explanation, a strong relationship between UEFM at T_1_ and T_2_ might explain why GE was not capable to explain additional variances. Taking NIHSS at T_1_ into account for the initial deficit, GE would show a significant association with UEFM at T_2_ across the complete cohort (Supplementary Table 2). Finally, *P*-values remained uncorrected for multiple comparisons in the regression analyses leading to good sensitivity at the cost of reduced specificity. This work has been designed as an exploratory re-analysis of two independent cohorts of stroke patients which have been already published.^29,30^ Hence, caution is advised when interpreting the present findings, prospective studies are needed to obtain further insights.

## Conclusion

This study provides empirical evidence that structural network efficiency as a graph-theoretical marker of large-scale network topology, quantified by diffusion MRI and individual whole-brain tractography early after stroke, relates to recovery. Notably, this contribution was only evident in severely but not mildly affected stroke patients. This suggests that the initial deficit might shape the dependency of recovery on global network topology after stroke.

## Supplementary Material

fcac049_Supplementary_Data

## Abbreviations

BI =: Barthel Index
DWI =: diffusion-weighted imaging
mRS =: modified Rankin Scale
NIHSS =: National Institutes of Health Stroke Scale
QSI =: q-space imaging
UEFM =: Upper-Extremity Score of the Fugl-Meyer Assessment
