# Supplementary material for: Brain network topology early after stroke relates to recovery
Source: Brain Commun. 2022 Feb 22;4(2):fcac049. doi: 10.1093/braincomms/fcac049 (PMC8905614; doi:10.1093/braincomms/fcac049)
Supplement: fcac049_Supplementary_Data [file fcac049_supplementary_data.pdf]

Supplemental Material

Brain network topology early after stroke relates to recovery

Paul R. Nemati[1], Winifried Backhaus[1], Jan Feldheim[1], Marlene Bönstrup[1,2], Bastian Cheng[1], Götz Thomalla[1], Christian Gerloff[1] and Robert Schulz[1]

[1] Department of Neurology, University Medical Center Hamburg-Eppendorf, 20246 Hamburg, Germany
[2] Department of Neurology, University Medical Center Leipzig, 04103 Leipzig, Germany

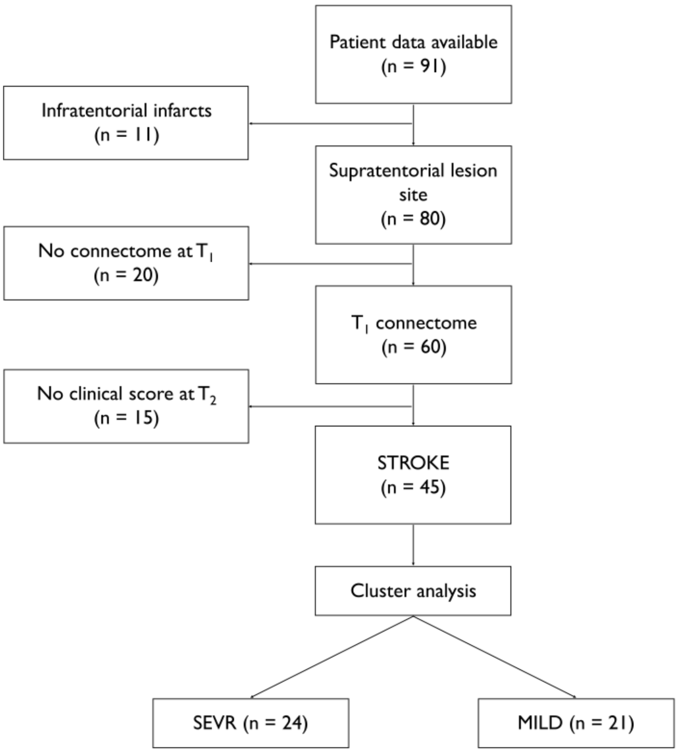

Supplemental Figure 1. Flow diagram of study inclusion.
Reasons for patient exclusion from the final study cohort are listed. Out of 91 available patient data, 45 were included in the analysis and differentiated by means of cluster analysis into two pre-specified subgroups; patients with severe (SEVR) and patients with rather mild-moderate initial deficits (MILD).

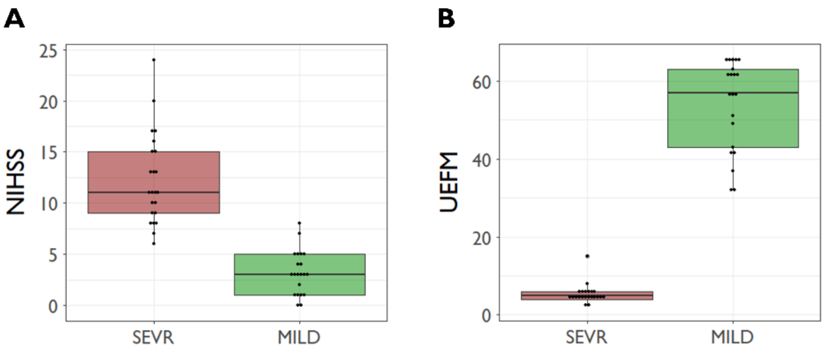

Supplemental Figure 2. Cluster allocation visualized for NIHSS and UEFM scores.

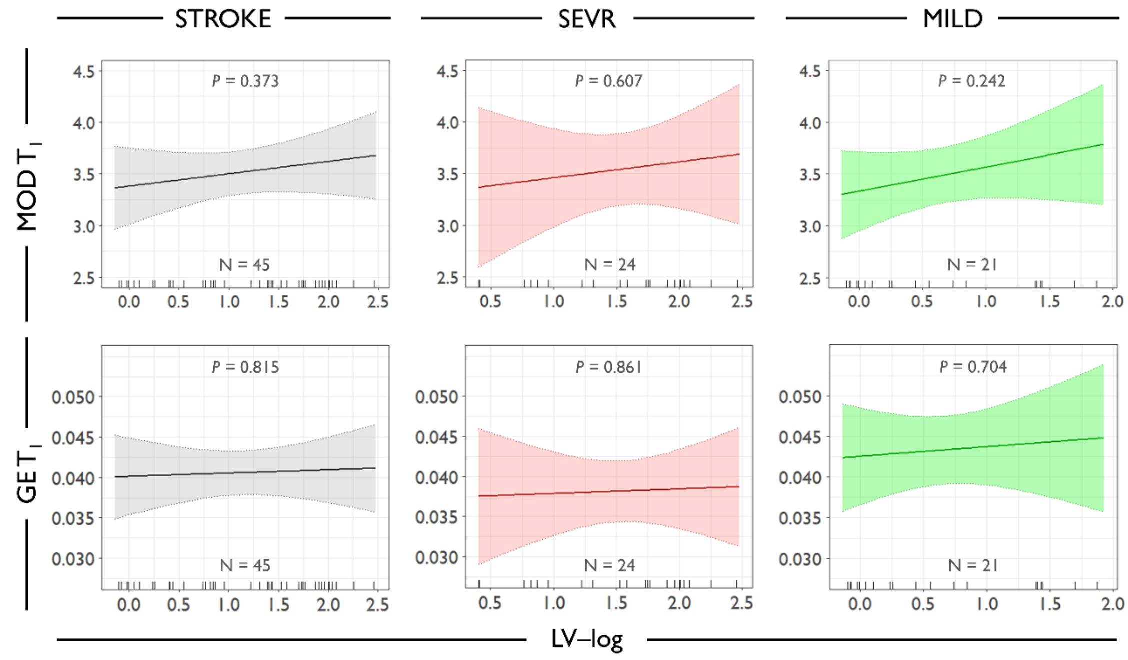

Supplemental Figure 3. Early structural network measures and stroke lesion volume.
Effect plots showing non-significant associations lesion volume (LV–log) with modularity (MOD) and global efficiency (GE) at T_1, respectively for STROKE, SEVR, and MILD. Models were adjusted for age. *P*-values are given for the predictor of interest LV–log. N Number of patients contributing to the model.

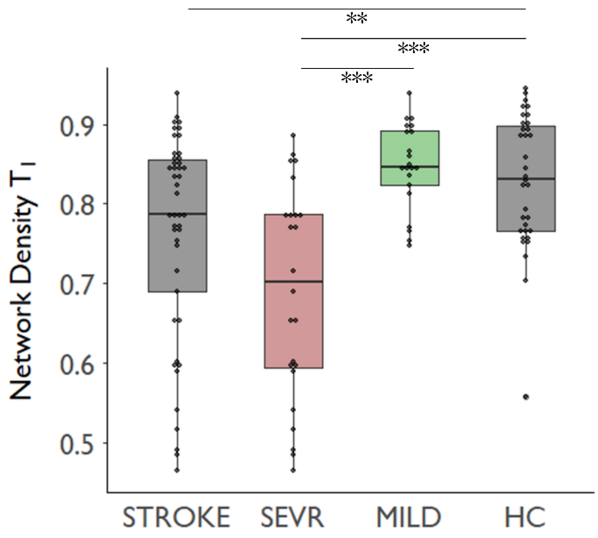

Supplemental Figure 4. Data distribution of network density
Boxplots depicting network density (D) for STROKE, SEVR, MILD and HC. Group comparisons were conducted by computing least-squares from linear models including GROUP and AGE with Tukey's test as the post-hoc analysis method. ** depicts *P*<0.01, *** *P*<0.001.

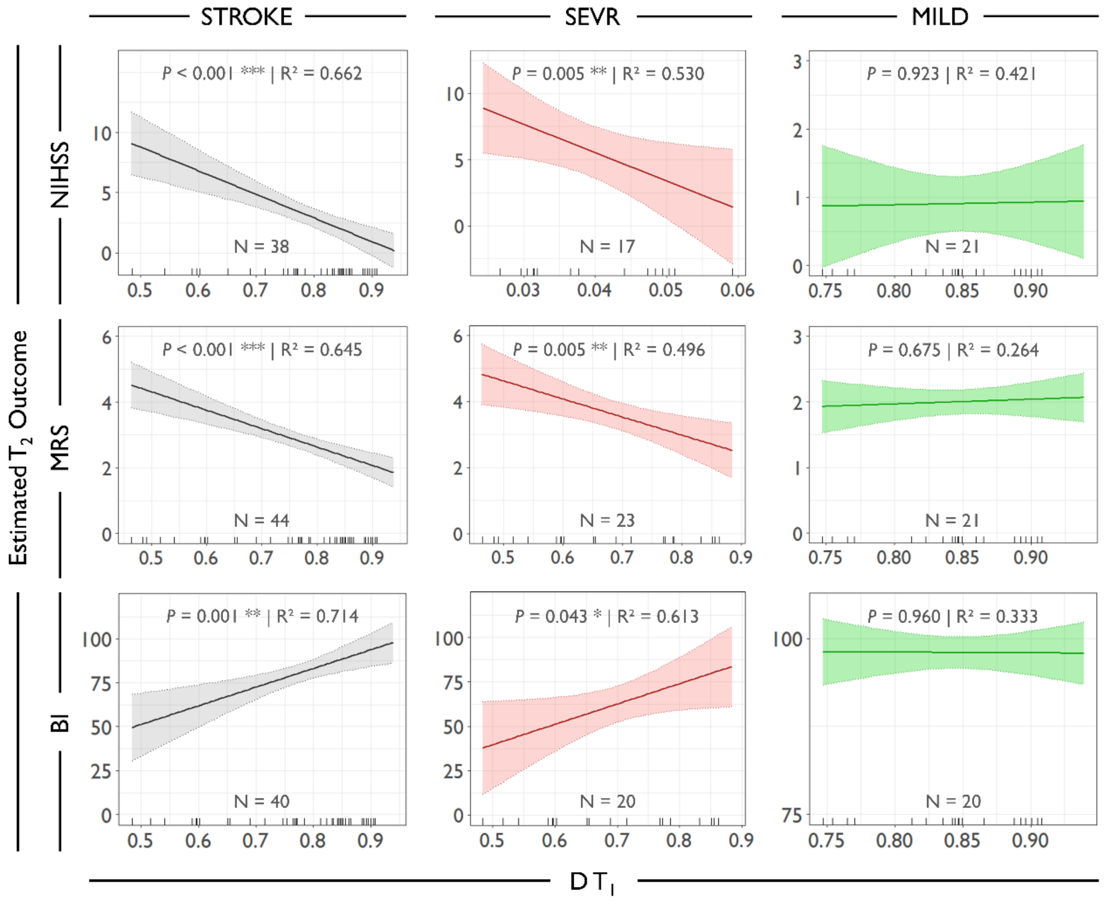

Supplemental Figure 5. Early structural network density and subsequent recovery after stroke.
Effect plots showing associations between early structural network density (D) at T_1 and the estimated outcome at T2 for all stroke patients (STROKE models without GROUP and interaction term). Effect plots are also given separately for SEVR and MILD. P-values are given for the predictor of interest D, multiple R^2 are given for the complete models. N Number of patients contributing to the model. * depicts *P*<0.05, ** *P*<0.01, *** *P*<0.001

	STROKE (1)	SEVR (2)	MILD (3)	HC (4)	1-4	2-4	3-4	2-3
	**Mean (95% CI)**	**Mean (95% CI)**	**Mean (95% CI)**	**Mean (95% CI)**	***P***	***P***	***P***	***P***
N	45	24	21	34	–	–	–	–
CC	0.0035 (0.0033-0.0038)	0.0036 (0.0033-0.0039)	0.0034 (0.0031-0.0038)	0.0039 (0.0036-0.0042)	0.060	0.410	0.139	0.795
CPL	1.24 (0.54–1.94)	1.66 (0.70-2.62)	0.75 (0-1.79)	0.64 (0-1.45)	0.270	0.246	0.986	0.418
D	0.76 (0.73–0.79)	0.70 (0.66-0.73)	0.83 (0.80-0.87)	0.83 (0.80-0.87)	0.004**	<0.001 ***	0.984	<0.001 ***

Supplemental Table 1. Group comparison of additional structural network parameters.
Estimated means with 95% confidence intervals (CI) are given for each group. X-Y indicates the pair of groups for comparison. *P*-values of post-hoc group comparisons between SEVR, MILD and HC were corrected for multiple testing using Tukey tests. Comparisons of network measures were adjusted for age. ** depicts $P<0.01$, *** $P<0.001$.
CC = clustering coefficient, CPL = characteristic path length, D = network density.

Outcome	Group	Predictor	Model Summary Coef.	*P*	F	R^2
NIHSS T_2	STROKE	GE	-0.61	0.002**	12.47	0.602
		GROUP		0.292		
		GE * GROUP		0.020*		
		NIHSS T_1	0.44	0.030*		
	SEVR	GE	-0.50	0.032*	4.56	0.395
		NIHSS T_1	0.38	0.091		
	MILD	GE	0.11	0.510	10.73	0.544
		NIHSS T_1	0.69	< 0.001***		
MRS T_2	STROKE	GE	-0.49	0.005**	14.54	0.599
		GROUP		0.213		
		GE * GROUP		0.040*		
		NIHSS T_1	0.44	0.021*		
	SEVR	GE	-0.38	0.019*	6.88	0.408
		NIHSS T_1	0.44	0.013*		
	MILD	GE	0.16	0.249	1.64	0.154
		NIHSS T_1	0.31	0.022*		
BI T_2	STROKE	GE	0.71	< 0.001***	19.97	0.689
		GROUP		0.535		
		GE * GROUP		0.007**		
		NIHSS T_1	-0.52	0.003**		
	SEVR	GE	0.52	0.002**	16.20	0.656
		NIHSS T_1	-0.54	0.002**		
	MILD	GE	0.12	0.609	0.73	0.075
		NIHSS T_1	-0.29	0.244		
UEFM T_2	STROKE	GE	0.24	0.014*#	42.28	0.701
		NIHSS T_1	-0.79	< 0.001***		

Supplemental Table 2. Early structural network efficiency and subsequent recovery after stroke.
Linear models correlating global efficiency (GE) at T_1 with clinical outcome at T_2. GE*GROUP interactions were evaluated for the whole stroke cohort (STROKE). Group-specific models were fit in the case of a significant interaction. Model predictors are derived from baseline models and model simplification (see Methods/Statistics). NIHSS T_1 was included as the initial deficit in all baseline models. Outcome and predictor values were z-standardized to enable comparability of coefficients. R^2 given as multiple R^2 of the complete model. * depicts $P<0.05$, ** $P<0.01$, *** $P<0.001$. # Model driven by one stroke patient (leave-one-out analysis: $P=0.15$).

Outcome	Group	Predictor	Model Summary Coef.	*P*	F	R^2
NIHSS T_2	STROKE	D	-0.69	<0.001***	20.06	0.709
		GROUP		0.028*		
		D * GROUP		0.028*		
		NIHSS T_1	0.23	0.177		
	SEVR	D	-0.71	0.005**	7.81	0.527
		NIHSS T_1	0.03	0.903		
	MILD	D	-0.02	0.900	10.27	0.533
		NIHSS T_1	0.74	<0.001***		
MRS T_2	STROKE	D	-0.63	<0.001***	21.55	0.689
		GROUP		0.034*		
		D * GROUP		0.044*		
		MRS T_1	0.23	0.091		
	SEVR	D	-0.59	0.005**	9.86	0.496
		MRS T_1	0.19	0.323		
	MILD	D	0.09	0.675	3.22	0.264
		MRS T_1	0.51	0.021*		
BI T_2	STROKE [#]	D	0.56	< 0.001***	25.37	0.744
		GROUP		0.068		
		D * GROUP		0.059 [#]		
		BI T_1	0.45	0.007**		
	SEVR	D	0.44	0.043*	13.48	0.613
		BI T_1	0.42	0.050		
	MILD	D	-0.01	0.960	4.25	0.333
		BI T_1	0.58	0.010**		
UEFM T_2	STROKE	D	0.21	0.013*	99.08	0.850
		UEFM T_1	0.79	< 0.001***		

Supplemental Table 3. Network density and subsequent recovery after stroke.
Linear models correlating network density (D) at T_1 with clinical outcome at T_2. D*GROUP interactions were evaluated for the whole stroke cohort (STROKE). Group-specific models were fit if the interaction was kept in the model during model simplification ([#]). Model predictors are derived from baseline models and model simplification (see Methods/Statistics). Outcome and predictor values were z-standardized to enable comparability of coefficients. R^2 given as multiple R^2 of the complete model. * depicts P<0.05, ** P<0.01, *** P<0.001.

Outcome	Group	Predictor	Model Summary			
			Coef.	*P*	F	R^2
NIHSS T_2	STROKE	MOD	0.11	0.396	13.88	0.442
		NIHSS T_1	0.67	<0.001***		
MRS T_2	STROKE #	MOD	0.30	0.029*	11.53	0.542
		GROUP		0.016*		
		MOD * GROUP		0.139 #		
		MRS T_1	0.34	0.036*		
BI T_2	STROKE	MOD	-0.12	0.242	32.35	0.636
		BI T_1	0.78	<0.001***		
UEFM T_2	STROKE	MOD	0.07	0.332	82.66	0.825
		UEFM T_1	0.91	<0.001***		

Supplemental Table 4. Early structural network modularity and subsequent recovery after stroke.
Linear models correlating modularity (MOD) at T_1 with clinical outcome at T_2. MOD*GROUP interactions were evaluated for the whole stroke cohort (STROKE). As none of these were significant (but kept in the final model during stepwise model simplification for MRS T_2 #), subgroup analyses were not conducted. When manually omitting MOD*GROUP and GROUP from the MRS model, the main effect for MOD would not be significant (*P*=0.171). Model predictors are derived from baseline models and model simplification (see Methods/Statistics). Outcome and predictor values were z-standardized to enable comparability of coefficients. R^2 given as multiple R^2 of the complete model. * depicts $P<0.05$, *** $P<0.001$.

Outcome	Group	Predictor	Model Summary			
			Coef.	*P*	F	R^2
NIHSS T_2	STROKE	CC	-0.19	0.131	15.33	0.467
		NIHSS T_1	0.69	<0.001***		
MRS T_2	STROKE	CC	0.01	0.907	14.08	0.407
		MRS T_1	0.64	<0.001***		
BI T_2	STROKE	CC	0.11	0.287	32.02	0.634
		BI T_1	0.78	<0.001***		
UEFM T_2	STROKE	CC	0.07	0.303	83	0.826
		UEFM T_1	0.91	<0.001***		

Supplemental Table 5. Early structural network clustering coefficient and subsequent recovery after stroke.
Linear models correlating clustering coefficient (CC) at T_1 with clinical outcome at T_2. CC*GROUP interactions were evaluated for the whole stroke cohort (STROKE). As none of these were significant, subgroup analyses were not conducted. Model predictors are derived from baseline models and model simplification (see Methods/Statistics). Outcome and predictor values were z-standardized to enable comparability of coefficients. R^2 given as multiple R^2 of the complete model.
*** depicts $P<0.001$.

Outcome	Group	Predictor	Model Summary Coef.	P	F	R^2
NIHSS T_2	STROKE	CPL	-0.16	0.224	14.57	0.454
		NIHSS T_1	0.70	<0.001***		
MRS T_2	STROKE	CPL	0.06	0.627	14.27	0.410
		MRS T_1	0.63	<0.001***		
BI T_2	STROKE	CPL	0.06	0.573	30.90	0.626
		BI T_1	0.80	<0.001***		
UEFM T_2	STROKE	CPL	-0.03	0.648	80.55	0.822
		UEFM T_1	0.90	<0.001***		

Supplemental Table 6. Early structural network characteristic path length and subsequent recovery after stroke.
Linear models correlating characteristic path length (CPL) at T_1 with clinical outcome at T_2. CPL*GROUP interactions were evaluated for the whole stroke cohort (STROKE). As none of these were significant, subgroup analyses were not conducted. Model predictors are derived from baseline models and model simplification (see Methods/Statistics). Outcome and predictor values were z-standardized to enable comparability of coefficients. R^2 given as multiple R^2 of the complete model.
*** depicts $P<0.001$.

ID	NIHSS subitems 1		2		4 - 6		8		9		10		11	
	T_1	T_2	T_1	T_2	T_1	T_2	T_1	T_2	T_1	T_2	T_1	T_2	T_1	T_2
4	1	0	0	0	7	1	1	0	2	0	1	0	1	0
5*	0	0	1	0	10	10	0	0	0	0	0	0	0	0
7*	2	2	0	0	8	10	0	1	1	1	0	1	0	0
8*	0	0	0	0	10	7	1	0	0	0	0	0	0	0
10*	0	0	0	0	8	2	0	0	0	0	0	0	0	0
11	1	0	2	0	8	11	1	1	0	0	1	1	2	1
12	0	0	2	0	8	2	2	1	0	0	1	0	2	0
13	0	0	1	1	9	9	2	1	0	0	1	0	2	2
14	0	0	0	0	4	1	1	0	1	1	1	1	0	0
16	0	0	0	0	10	3	1	0	0	0	1	0	1	0
17	0	0	0	0	10	5	2	0	0	0	1	0	0	0
19	0	0	0	0	7	4	1	1	1	0	1	1	0	0
20	0	0	0	0	8	3	1	0	0	0	0	0	0	0
21	0	0	0	0	8	2	0	0	0	0	0	0	0	0
22	0	0	0	0	7	4	0	0	0	0	1	0	0	0
23	0	0	0	0	6	4	1	0	0	0	0	0	0	0
24	0	0	0	0	6	3	0	0	0	0	0	0	0	0

Supplemental Table 7. T_1 and T_2 values for subitems of the National Institutes of Health Stroke Scale (NIHSS) for SEVR.
Baseline and outcome NIHSS subitem scores are given individually for severely affected stroke patients. Assessments took place in the acute stage (T_1) 3-14 days after the event and in the late subacute stage (T_2) either three or six months (depicted by * at respective ID) after stroke. NIHSS = National Institutes of Health Stroke Scale. The following subitems were explored: 1 Consciousness, 2 Best gaze, 4-6 Motor scores with facial palsy, motor arm and motor leg, 8 Sensory functions, 9 Best language, 10 Dysarthria, 11 Extinction and inattention. Subitems 3 and 7 are not given as all patients scored zero on these items.

NIHSS subitem	Predictor	Model Summary			
		Coef.	*P*	F	R^2
1	GE	-0.05	0.759	13.38	0.657
	NIHSS 1 T_1	0.80	<0.001***		
2	GE	-0.33	0.198	1.39	0.165
	NIHSS 2 T_1	0.19	0.459		
4 - 6	GE	-0.49	0.026*	5.97	0.460
	NIHSS 4-6 T_1	0.49	0.025*		
8	GE	-0.12	0.643	1.07	0.133
	NIHSS 8 T_1	0.34	0.189		
9	GE	-0.39	0.088	3.91	0.358
	NIHSS 9 T_1	0.48	0.041*		
10	GE	-0.38	0.159	1.63	0.189
	NIHSS 10 T_1	0.38	0.165		
11	GE	-0.38	0.040*	10.73	0.605
	NIHSS 11 T_1	0.65	0.002**		

Supplemental Table 8. Early structural network efficiency and subsequent recovery after stroke measured by subitems of the National Institutes of Health Stroke Scale in SEVR.
Linear models correlating global efficiency (GE) at T_1 with subitem values of the NIHSS at T_2 in the SEVR subgroup. Subitems 4-6 were combined to form one subitem of motor impairment. As all participants had values of zero for subitem 3 and 7, models could not be fit for these items. Model predictors are chosen in analogy to main table 4. Outcome and predictor values were z-standardized to enable comparability of coefficients. R^2 given as multiple R^2 of the complete model.
* depicts $P<0.05$, ** $P<0.01$, *** $P<0.001$.
